# Dehydroepiandrosterone-induced activation of mTORC1 and inhibition of autophagy contribute to skeletal muscle insulin resistance in a mouse model of polycystic ovary syndrome

**DOI:** 10.18632/oncotarget.24190

**Published:** 2018-01-12

**Authors:** Xi Song, Qiyang Shen, Liting Fan, Qiuxiao Yu, Xiao Jia, Yu Sun, Wenpei Bai, Jihong Kang

**Affiliations:** ^1^ Department of Physiology and Pathophysiology, School of Basic Medical Sciences, Peking University Health Science Center, Beijing, China; ^2^ Institute of Infectious Diseases, Beijing Ditan Hospital, Capital Medical University, Beijing, China; ^3^ Department of Obstetrics and Gynecology, Beijing Shijitan Hospital, Beijing, China

**Keywords:** polycystic ovary syndrome (PCOS), dehydroepiandrosterone (DHEA), mTOR, autophagy, skeletal muscle insulin resistance

## Abstract

Polycystic ovary syndrome (PCOS) is the most common endocrinopathy in women of reproductive age and also an important metabolic disorder associated with insulin resistance (IR). Hyperandrogenism is a key feature of PCOS. However, whether hyperandrogenism can cause IR in PCOS remains largely unknown. The mammalian target of rapamycin complex 1 (mTORC1) and its regulated autophagy are closely associated with IR. In the present study, we investigated the role of mTORC1-autophagy pathway in skeletal muscle IR in a dehydroepiandrosterone (DHEA)-induced PCOS mouse model. DHEA-treated mice exhibited whole-body and skeletal muscle IR, along with the activated mTORC1, repressed autophagy, impaired mitochondria, and reduced plasma membrane glucose transporter 4 (GLUT4) expression in skeletal muscle of the mice. In cultured C2C12 myotubes, treatment with high dose testosterone activated mTORC1, reduced autophagy, impaired mitochondria, decreased insulin-stimulated glucose uptake, and induced IR. Inhibition of mTORC1 or induction of autophagy restored mitochondrial function, up-regulated insulin-stimulated glucose uptake, and increased insulin sensitivity. On the contrary, inhibition of autophagy exacerbated testosterone-induced impairment. Our findings suggest that the mTORC1-autophagy pathway might contribute to androgen excess-induced skeletal muscle IR in prepubertal female mice by impairing mitochondrial function and reducing insulin-stimulated glucose uptake. These data would help understanding the role of hyperandrogenism and the underlying mechanism in the pathogenesis of skeletal muscle IR in PCOS.

## INTRODUCTION

Polycystic ovary syndrome (PCOS) is the most common endocrinopathy characterized by menstrual irregularity, hyperandrogenism, and polycystic ovarian morphologic features. It affects 6–10% of women of reproductive age [1] and is the most common cause of anovulatory infertility. In most cases, PCOS also involves metabolic abnormalities, such as obesity and dyslipidemia. One of its most important clinical features is insulin resistance (IR), which has been associated with increased risk for type 2 diabetes mellitus (T2D) [2, 3]. IR occurs in 44–85% of women of PCOS [4] and appears to be independent of obesity in PCOS patients. The pathogenesis of IR in PCOS patients remains unknown. Skeletal muscle is responsible for approximately 80% of insulin-stimulated glucose uptake in the whole body. Previous studies demonstrated that PCOS patients have impaired insulin signaling in skeletal muscle, including reduced insulin-stimulated phosphorylation of protein kinase B (Akt) and its 160 kDa substrate (AS160) [5], and increased phosphorylation of insulin receptor substrate 1 (IRS-1) [6].

The mammalian target of rapamycin (mTOR) is a central nutrient sensor. It assembles into two distinct multiprotein complexes, termed mTOR complex 1 (mTORC1) and mTORC2. Bentzinger and colleagues have shown the critical role for mTORC1 in muscle function, mitochondrial biogenesis, and metabolic properties [7]. The mTORC1 is also the well-characterized regulator of autophagy. Inhibition of mTORC1 activates autophagy, which is required for the clearance of dysfunctional cellular components. Autophagy is essential for maintaining cellular homeostasis and survival. Previous studies have revealed that autophagy regulates muscle mass [8] and autophagy deficiency in skeletal muscle leads to protection from diet-induced obesity and insulin resistance [9]. However, it is unknown whether mTORC1 and its regulated autophagy play a role in skeletal muscle IR in PCOS.

We have previously reported the whole-body IR in dehydroepiandrosterone (DHEA)-induced PCOS mice. In this study, we aimed to investigate the possible role for mTORC1 and autophagy in skeletal muscle IR in a PCOS mouse model induced by DHEA.

## RESULTS

### Whole-body and skeletal muscle insulin resistance in DHEA mice

**Figure 1:**
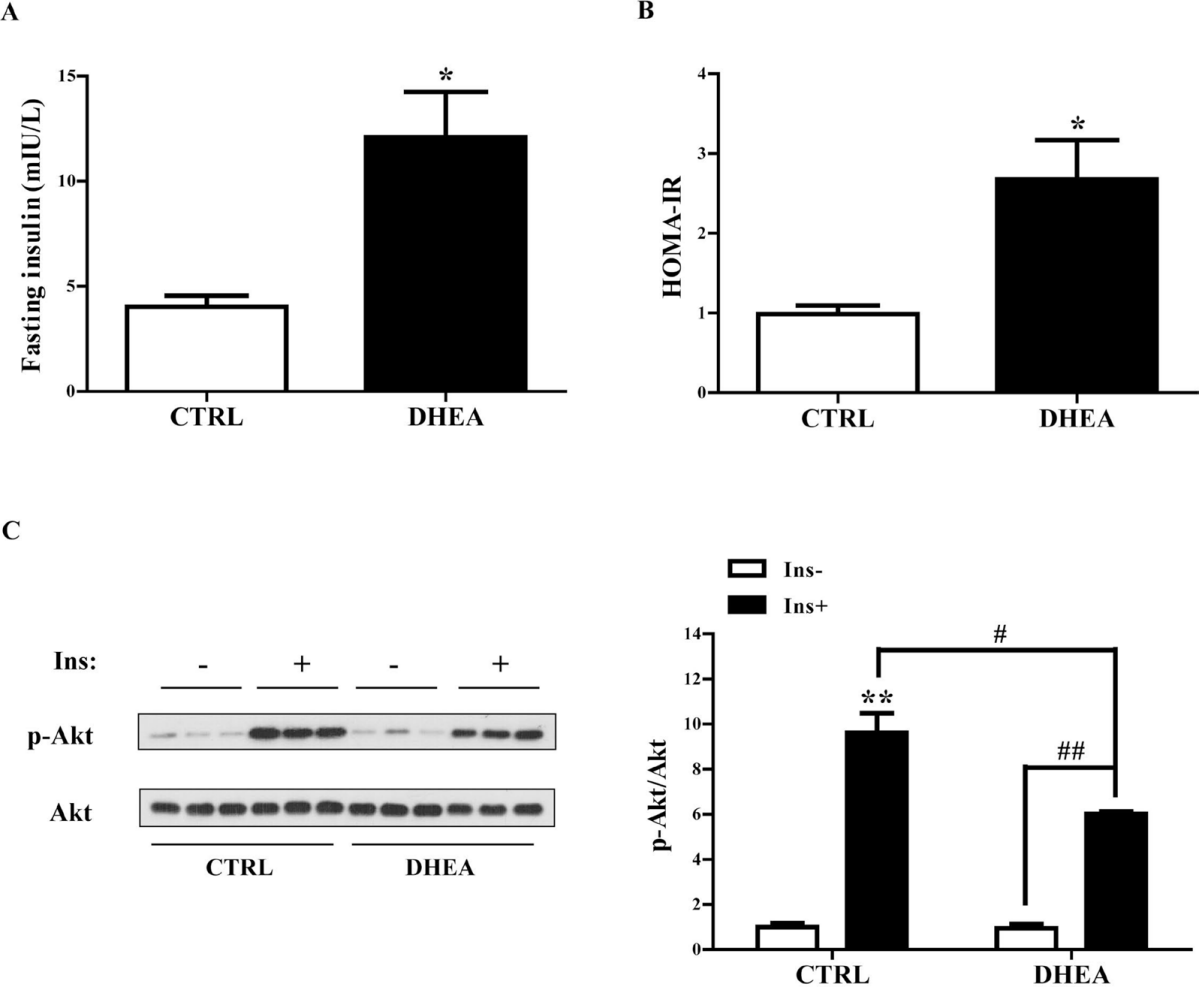
Reduced insulin sensitivity in DHEA-induced PCOS mice Mice were fasted overnight. (**A**) Fasting serum insulin levels. (**B**) HOMA-IR index. (**C**) Mice were injected with saline or 1.0 IU/kg b.w. insulin. Insulin-signaling assay showed baseline and insulin-stimulated p-Akt in the skeletal muscle. Data are represented as mean ± SEM. ^*^P < 0.05, ^**^P < 0.01, vs control; ^#^*P* < 0.05, ^##^*P* < 0.01. *n* = 6/group.

We have previously shown the whole-body IR in DHEA-induced PCOS mice by Insulin Tolerance Test (ITT) [10]. In the present work, we evaluated the HOMA-IR index of the animals. Fasting glucose levels were similar between DHEA and control mice (data not shown). As expected, fasting serum insulin levels were markedly increased in DHEA mice (*P* < 0.05) (Figure 1A). Accordingly, HOMA-IR was significantly greater in DHEA mice than in controls (*P* < 0.05) (Figure 1B), confirming whole-body IR in DHEA-induced PCOS mice.

To evaluate insulin sensitivity in the skeletal muscle, insulin signaling assay was performed. After the treatments for 20 days, the mice were fasted overnight and then were treated with insulin or saline for 15 min. Skeletal muscle tissues were harvested. Results from Western blot analysis revealed that p-Akt levels were increased in skeletal muscle of the two groups of mice following insulin treatment (*P* < 0.01 and *P* < 0.01, respectively) (Figure 1C). However, DHEA mice exhibited blunted phosphorylation of Akt after insulin administration compared with controls (*P* < 0.05) (Figure 1C), indicating IR in the skeletal muscle of DHEA mice.

### Increased phosphorylation of mTORC1 in the skeletal muscle of DHEA mice

**Figure 2:**
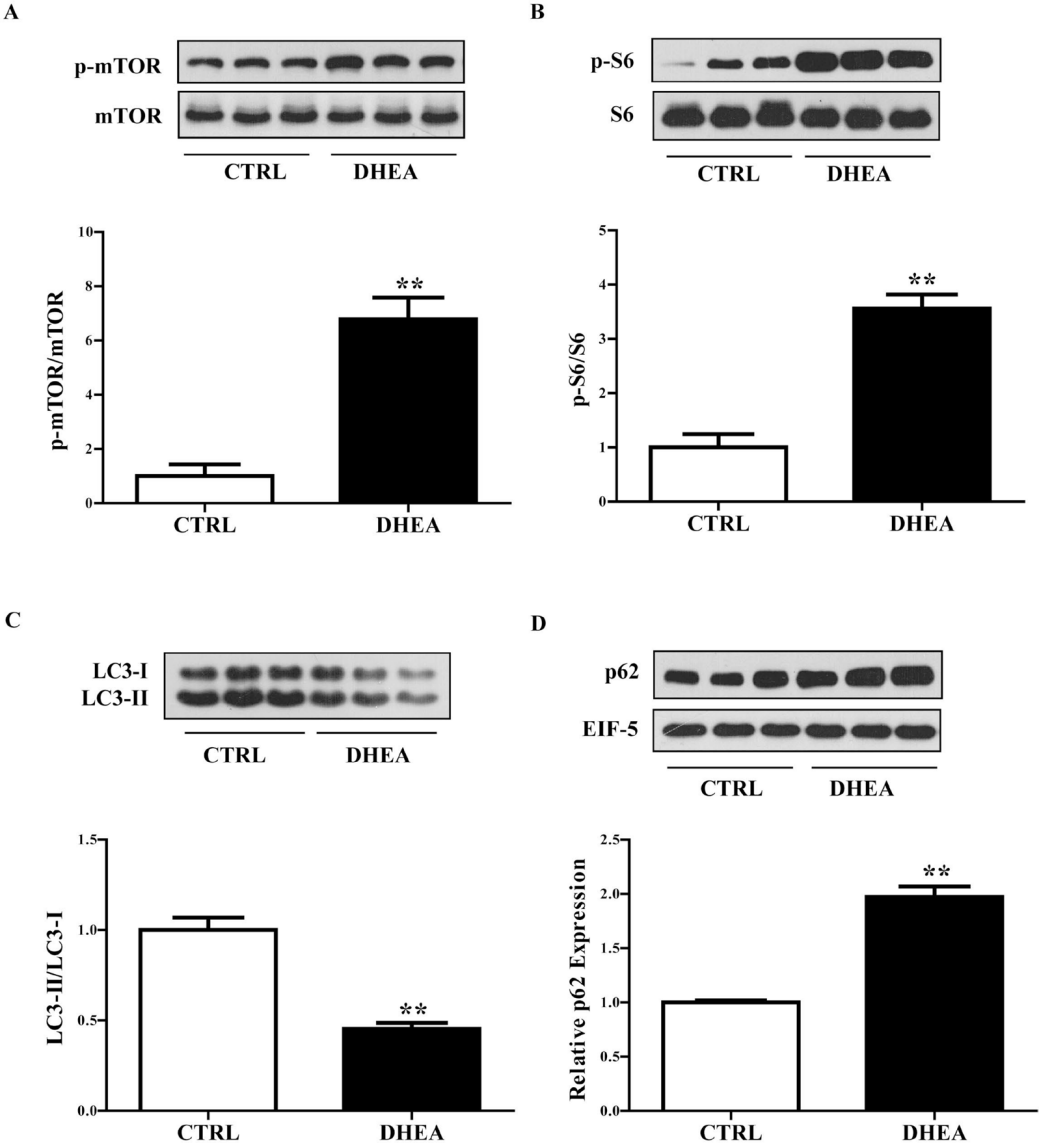
Expression of mTOR- and autophagy-related genes in the skeletal muscle of the mice After the treatments for 20 days, mice were killed and skeletal muscle tissues were collected. Representative Western blots and densitometry quantification of (**A**) p-mTOR/mTOR, (**B**) p-S6/S6, (**C**) LC3-II/LC3-I, (**D**) p62 and EIF-5. EIF-5 was used as a control. Data are expressed as mean ± SEM. ^*^P < 0.05, ^**^P < 0.01, vs control; ^#^*P* < 0.05, ^##^*P* < 0.01. *n* = 6/group.

After the treatments for 20 days, six mice per group were killed and skeletal muscles were collected. Data from Western blot analysis showed that phosphorylation of mTOR was significantly increased in DHEA mice compared with controls (*P* < 0.01) (Figure 2A). S6 is a main downstream target of S6 Kinase 1 (S6K1), a well-characterized substrate of mTORC1. The phosphorylated S6 is a surrogate marker of mTORC1 activation. Similar to mTOR, the phosphorylation of S6 was also markedly elevated in DHEA mice compared with controls (*P* < 0.01) (Figure 2B), indicating that DHEA treatment activated mTORC1 in the skeletal muscle of the mice.

### Reduced autophagy in the skeletal muscle of DHEA mice

To investigate whether autophagy is changed in the skeletal muscle of the mice, the expression of LC3 and p62, two widely used markers to monitor autophagy, was detected. DHEA mice exhibited significantly lower LC3-II/LC3-I ratios (*P* < 0.01) (Figure 2C) and higher p62 levels than controls (*P* < 0.01) (Figure 2D), suggesting decreased autophagy in the skeletal muscle of DHEA mice.

### Mitochondrial impairment in the skeletal muscle of DHEA mice

**Figure 3:**
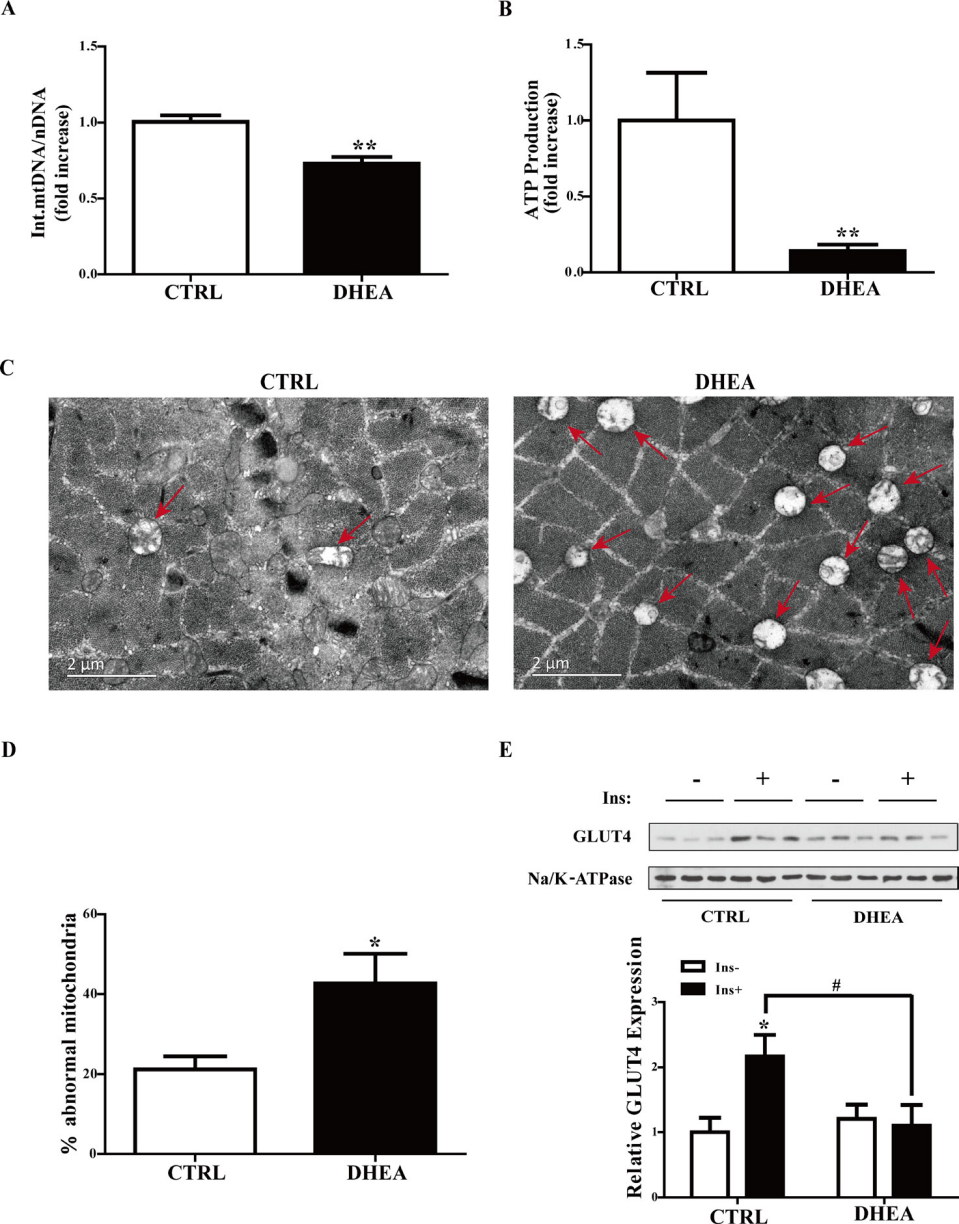
Effect of the treatments on integrated mitochondrial DNA copy number and mitochondrial function in the skeletal muscle of the mice (**A**) Ratio of integrated mitochondrial to nuclear DNA number. (**B**) Normalized ATP production. *n* = 6/group. (**C**) Representative TEM image of skeletal muscle samples from the mice. Arrows denote swollen mitochondria. Scale bar = 2 µm. (**D**) Statistical analysis of the percentage of swollen mitochondria. *n* = 4/group. (**E**) Representative Western blots and densitometry quantification of plasma membrane GLUT4. Na/K-ATPase served as a loading control of plasma membrane protein. Data are expressed as mean ± SEM. ^*^P < 0.05, ^**^P < 0.01, vs control; ^#^*P* < 0.05, ^##^*P* < 0.01. GLUT4: glucose transporter 4.

Mitochondria are central to muscle metabolism. We therefore studied the integrated mitochondrial DNA copy number and the mitochondrial function in the skeletal muscle of the mice. ND4 is a mitochondrial DNA (mtDNA) encoding one subunit of the NADH ubiquinone oxidoreductase which is complex I of the mitochondrial respiratory chain [11]. The ND4 gene is often deleted. The mtDNA deletions are frequently reported in diseases and aging. Therefore the ratio of ND4 to nuclear DNA Lpl is used to indicate the integrated mtDNA copy number. As illustrated in Figure 3A, the ratio of integrated mtDNA to nDNA was significantly lower in DHEA mice than in controls (*P* < 0.01), suggesting that DHEA treatment reduced the integrated mitochondrial number in skeletal muscle of the mice.

To investigate whether mitochondrial function was changed, ATP contents of skeletal muscle tissues were measured. ATP production was significantly lower in DHEA mice than in controls (*P* < 0.01) (Figure 3B), indicating that DHEA treatment impaired mitochondrial function in the skeletal muscle of the mice.

We next examined whether there were the pathological ultrastructure changes of mitochondria in the skeletal muscle tissues. Results from TEM showed that there were no obvious changes in organelles except mitochondria between the two groups of mice. In DHEA mice, there were much more abnormal mitochondria which presented swollen appearance and the presence of intramitochondrial vacuoles (Figure 3C and 3D), indicating that the number of damaged mitochondria was significantly increased in these animals.

Collectively, these data suggested that DHEA treatment led to mitochondrial impairment in the skeletal muscle.

### Decreased plasma membrane glucose transporter 4 (GLUT4) expression in the skeletal muscle of DHEA mice

Insulin-stimulated glucose uptake in skeletal muscle is regulated by GLUT4. In skeletal muscle, insulin induces GLUT4 translocation from intracellular storage compartments to the plasma membrane for glucose uptake. We thus measured GLUT4 expression on the plasma membrane. As shown in Figure 3E, plasma membrane GLUT4 expression was significantly increased following insulin treatment in controls (*P* < 0.05). In DHEA mice, there was no apparent difference in membrane GLUT4 levels in the presence or absence of insulin administration, suggesting that DHEA treatment reduced insulin-stimulated GLUT4 translocation in the skeletal muscle of the mice.

### Increased phosphorylation of mTORC1 and decreased autophagy by the treatment of testosterone (T) in C2C12 cells

**Figure 4:**
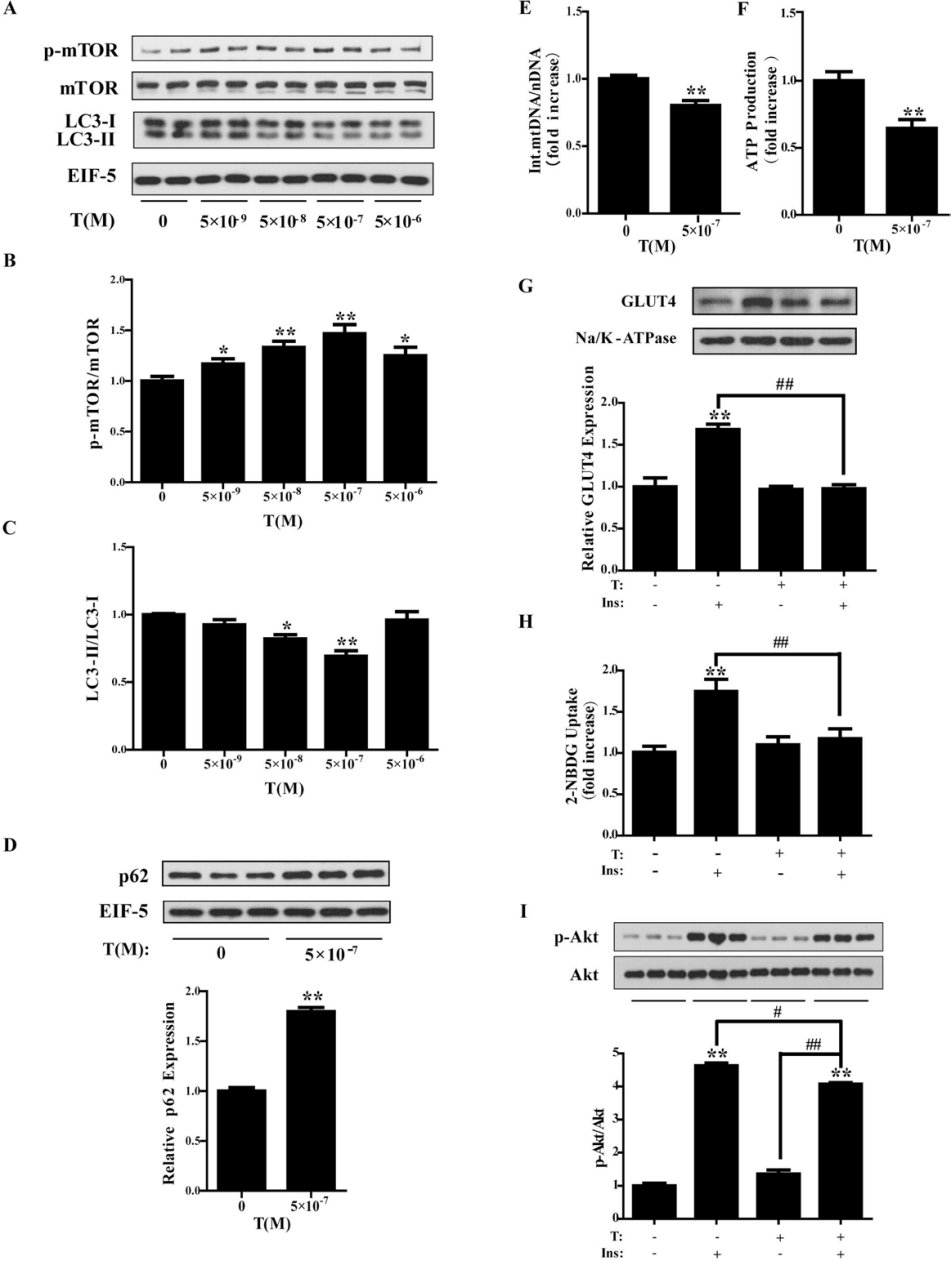
Treatment of testosterone in C2C12 cells (**A**) Differentiated C2C12 cells were treated with different concentrations of testosterone (T) (0, 5 × 10^-9^, 5 × 10^-8^, 5 × 10^-7^ and 5 × 10^-6^ M) for 24h. (A) Representative Western blots and densitometry quantification of (**B**) p-mTOR/mTOR and (**C**) LC3-II/LC3-I. (**D**) Differentiated C2C12 cells were treated with T (0, 5 × 10^-7^ M) for 24h. Representative Western blots and densitometry quantification of p62. EIF-5 was used as a control. (**E**) Ratio of integrated mitochondrial to nuclear DNA number. (**F**) Normalized ATP production. (**G**) Representative Western blots and densitometry quantification of plasma membrane GLUT4. Na/K-ATPase served as a loading control of plasma membrane protein. (**H**) 2-NBDG glucose uptake assay. (**I**) Insulin-signaling assay. Data are expressed as mean ± SEM. ^*^P < 0.05, ^**^P < 0.01, vs control; ^#^*P* < 0.05, ^##^*P* < 0.01. *n* = 6/group. T: testosterone. GLUT4: glucose transporter 4.

To study the underlying mechanism, C2C12 cells were cultured and treated with different concentrations of T (0, 5 × 10^-9^, 5 × 10^-8^, 5 × 10^-7^, and 5 × 10^-6^ M) for 24 h. As illustrated in Figure 4A and 4B, T significantly increased the phosphorylation of mTOR in a dose dependent manner when the concentrations of T were 5 × 10^-9^, 5 × 10^-8^, and 5 × 10^-7^ M (*P* < 0.05, *P* < 0.01, and *P* < 0.01, respectively). Treatment of T markedly reduced the ratio of LC3-II/LC3-I at 5 × 10^-8^ and 5 × 10^-7^ M (*P* < 0.05 and *P* < 0.01, respectively) (Figure 4C). No apparent effect on LC3-II/LC3-I was observed when T was at 5 × 10^-9^ or 5 × 10^-6^ M. Because of the remarkable effect of T at 5 × 10^-7^ M on the up-regulation of p-mTOR/mTOR and down-regulation of LC3-II/LC3-I, which mimicked the *in vivo* situations, the concentration of 5 × 10^-7^ M was thus chosen. We next treated C2C12 cells with T at 5 × 10^-7^ M in a time-dependent experiment. The p-mTOR/mTOR ratio was significantly higher when cells were treated for 24 h than for 6 or 12 h (data not shown). Therefore, C2C12 cells were treated with T at 5 × 10^-7^ M for 24 h in the later experiments. The C2C12 cells were then treated with 5 × 10^-7^ M of T for 24 h. There was a significant increase in p62 levels (*P* < 0.01) (Figure 4D), indicating the decreased autophagy by T treatment.

### Decreased integrated mtDNA copy number and ATP production by T treatment in C2C12 cells

C2C12 cells were treated with T (0, 5 × 10^-7^ M) for 24 h. The cells were then collected for DNA extraction. The ratio of ND4 to Lpl was significantly lower after T treatment than controls (Figure 4E), suggesting the reduced number of the integrated mitochondria by T treatment. ATP production was also measured. T significantly reduced ATP production (Figure 4F), suggesting the impaired mitochondrial function of the cells by T treatment.

### Decreased membrane GLUT4 expression and glucose uptake of C2C12 cells by T treatment

To investigate whether T affects glucose uptake, the membrane GLUT4 expression was measured. Cells were treated with T (0, 5 × 10^-7^ M) for 24 h, followed by the treatment with insulin (0, 100 nM) for 5 min. Then the cells were collected for plasma membrane protein extraction. Western blot analysis showed the significantly increased membrane GLUT4 levels by insulin stimulation. The pre-treatment of cells with T, however, markedly decreased insulin-induced GLUT4 expression (*P* < 0.01) (Figure 4G), indicating the reduced GLUT4 translocation by T treatment.

Glucose uptake was measured in C2C12 cells with the 2-NBDG commercial kit. As shown in Figure 4H, treatment of the cells with insulin significantly increased glucose uptake (*P* < 0.01). Treatment with T alone did not have any obvious effects on glucose uptake compared with control cells. The insulin+T treatment, however, remarkably decreased glucose uptake compared with insulin treatment alone (*P* < 0.01), indicating that T reduced insulin-stimulated glucose uptake.

### Insulin resistance of C2C12 cells induced by T treatment

We next investigated insulin sensitivity in C2C12 cells with T treatment. Cells were treated with T (0, 5 × 10^-7^ M) for 24 h, followed by the treatment with insulin (0, 100 nM) for 5 min. Then the cells were collected for protein extraction. Western blot analysis showed the significantly increased phosphorylation of Akt by insulin stimulation in the presence or absence of T (*P* < 0.01 and *P* < 0.01, respectively) (Figure 4I). However, cells treated with insulin+T exhibited blunted phosphorylation of Akt compared with cells treated with insulin alone, indicating insulin resistance of the cells induced by T (*P* < 0.05). It is interesting to note that treatment of the cells with T alone did not affect the phosphorylation of Akt compared with the control cells.

### Effects of rapamycin on T-treated C2C12 cells

**Figure 5:**
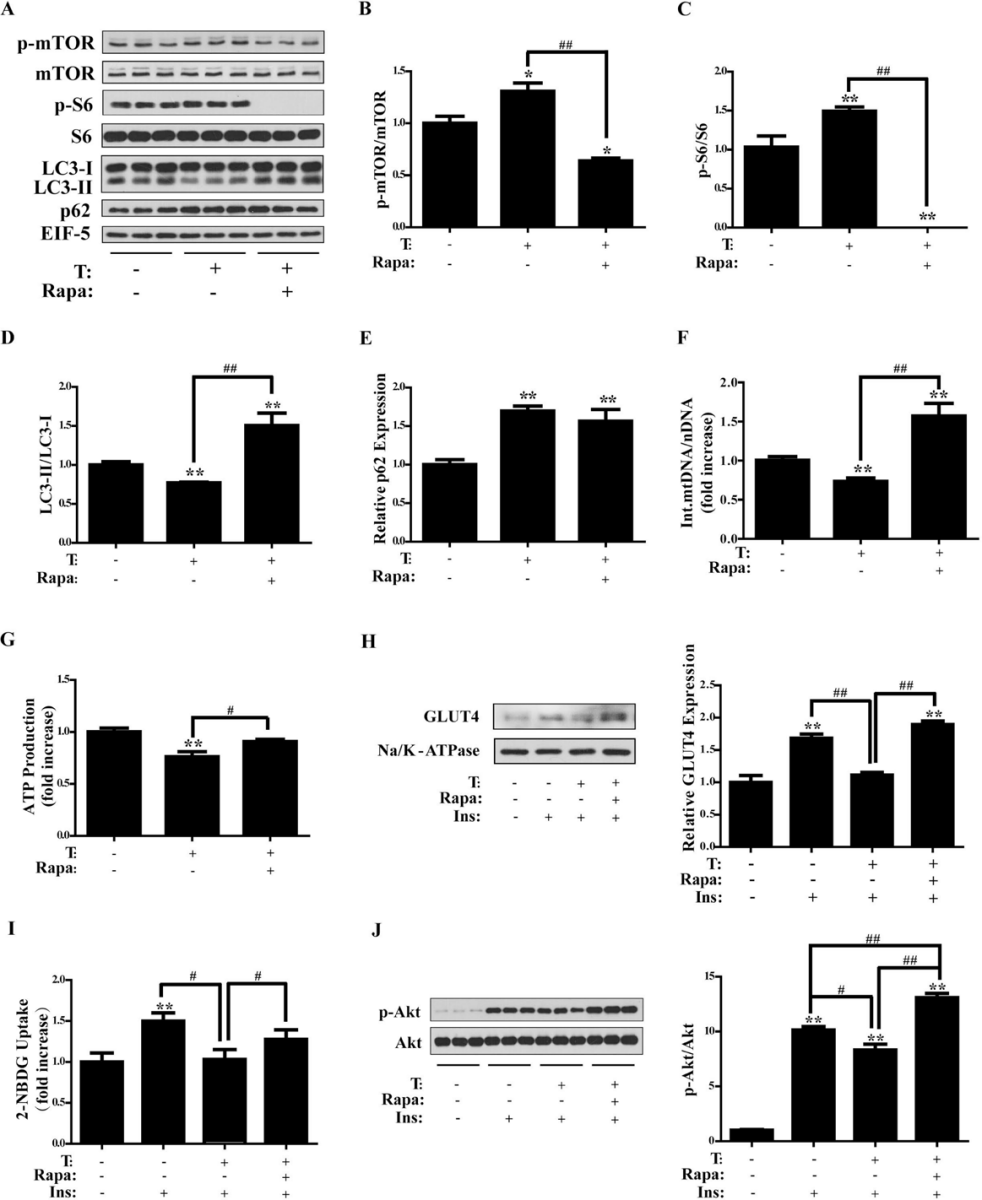
Effects of rapamycin on testosterone-treated C2C12 cells Differentiated C2C12 cells were treated with T (0, 5 **×** 10^-7^ M) for 24 h. During the last 2 h, rapamycin (0, 1 μM) was added and co-cultured with T. (**A**) Representative Western blots and densitometry quantification of (**B**) p-mTOR/mTOR, (**C**) p-S6/S6, (**D**) LC3-II/LC3-I, and (**E**) p62. EIF-5 was used as a control. (**F**) Ratio of integrated mitochondrial to nuclear DNA number. (**G**) Normalized ATP production. (**H**) Representative Western blots and densitometry quantification of plasma membrane GLUT4. Na/K-ATPase served as a loading control of plasma membrane protein. (**I**) 2-NBDG glucose uptake assay. (J) Insulin-signaling assay. Data are expressed as mean ± SEM. ^*^P < 0.05, ^**^P < 0.01, vs control; ^#^*P* < 0.05, ^##^*P* < 0.01. *n* = 6/group. T: testosterone, Rapa: rapamycin. GLUT4: glucose transporter 4.

Rapamycin is an inhibitor of mTORC1 and also an inducer of autophagy. To investigate the possible role for mTORC1 in T-induced insulin resistance, rapamycin was used in combination with T. Briefly, C2C12 cells were treated with T (0, 5 × 10^-7^ M) for 24 h. In the last 2 h, rapamycin (0, 1 µM) was added into the cell culture medium and co-cultured with T for 2 h. Then the cells were collected for protein assay. Results from Western blot showed that T+rapamycin significantly reduced phosphorylation of mTOR and S6 compared with T treatment alone (*P* < 0.01 and *P* < 0.01, respectively), confirming the inhibition of mTORC1 by rapamycin (Figure 5A, 5B and 5C). Rapamycin is also an inducer of autophagy. As expected, LC3-II/LC3-I ratio was markedly higher in T+ rapamycin group than in T and control groups (*P* < 0.01 and *P* < 0.01, respectively) (Figure 5D), indicating the up-regulated autophagy by rapamycin treatment. Both T and T+rapamycin treatments significantly induced the expression of p62 (*P* < 0.01 and *P* < 0.01, respectively) (Figure 5E). However, there was no difference in p62 expression between T and T+rapamycin groups. These data indicated that rapamycin increased LC3-II formation, but did not change p62 levels.

Compared with T treatment alone, T+rapamycin significantly increased the integrated mtDNA copy number (*P* < 0.01) (Figure 5F). ATP production was also measured. The cells were treated as described above and then were collected for ATP measurement. Compared with T treatment alone, T+rapamycin significantly increased ATP production (*P* < 0.05) (Figure 5G). There was no obvious difference between T+rapamycin and control groups. These data suggested the increased integrated mtDNA copy number and the restored mitochondrial function of cells by rapamycin treatment.

Likewise, plasma membrane GLUT4 expression and glucose uptake were measured to assess the effect of rapamycin on T-treated cells. C2C12 cells were treated with T (0, 5 × 10^-7^ M) or T+rapamycin as described above. Western blot analysis showed that plasma membrane GLUT4 expression was significantly higher in insulin+T+rapamycin group than in insulin+T group (*P* < 0.01) (Figure 5H), suggesting that rapamycin increased T-reduced membrane GLUT4 levels. Similarly, glucose uptake was significantly increased in insulin+T+rapamycin group compared with insulin+T group (*P* < 0.05) (Figure 5I). There was a trend for glucose uptake to be lower in insulin+T+rapamycin than in insulin group. However, there was no statistical difference between these two groups, indicating that rapamycin restored T-reduced glucose uptake in cells.

We then investigated insulin sensitivity of the cells. Western blot analysis showed the significantly reduced phosphorylation of Akt by insulin+T treatment compared with insulin group (*P* < 0.05) and the significantly increased phosphorylation of Akt by insulin+T+rapamycin compared with insulin+T treatment (*P* < 0.01) (Figure 5J). The p-Akt/Akt ratio was markedly greater in insulin+T+rapamycin group than in insulin group (*P* < 0.01). These data suggested that treatment of rapamycin restored T-reduced insulin sensitivity of the cells.

### Effects of leucine on T-treated C2C12 cells

**Figure 6:**
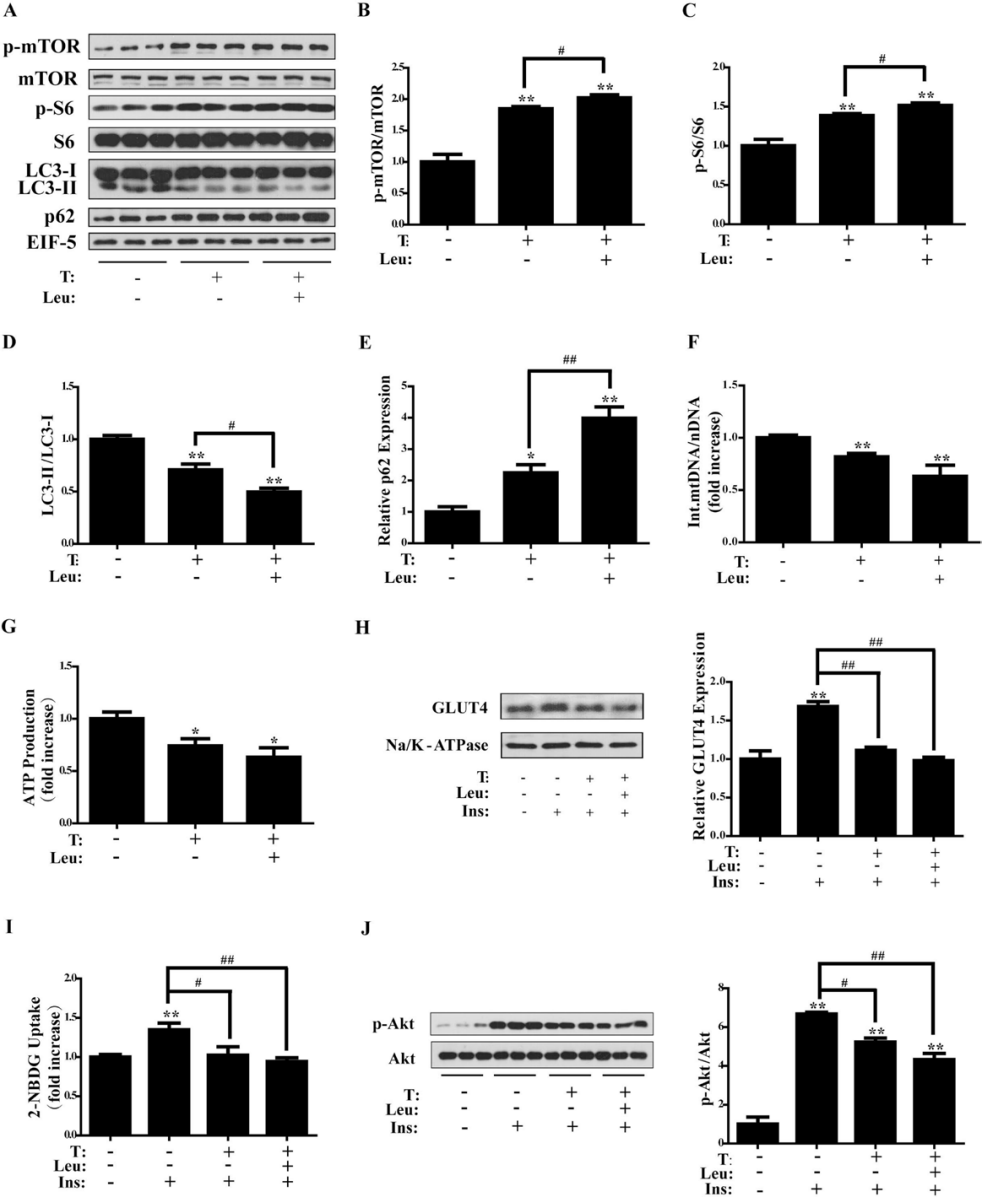
Effects of leucine on testosterone-treated C2C12 cells Differentiated C2C12 cells were pre-treated with leucine (0, 10 mM) for 2 h, followed by the treatment with T (0, 5 × 10^-7^ M) for 24 h. (**A**) Representative Western blots and densitometry quantification of (**B**) p-mTOR/mTOR, (**C**) p-S6/S6, (**D**) LC3-II/LC3-I, and (**E**) p62. EIF-5 was used as a control. (**F**) Ratio of integrated mitochondrial to nuclear DNA number. (**G**) Normalized ATP production. (**H**) Representative Western blots and densitometry quantification of plasma membrane GLUT4. Na/K-ATPase served as a loading control of plasma membrane protein. (**I**) 2-NBDG glucose uptake assay. (**J**) Insulin-signaling assay. Data are expressed as mean ± SEM. ^*^P < 0.05, ^**^P < 0.01, vs control; ^#^*P* < 0.05, ^##^*P* < 0.01. *n* = 6/group. T: testosterone, Leu: leucine. GLUT4: glucose transporter 4.

Leucine is an agonist of mTOR. To further investigate the possible role for mTOR in T-induced insulin resistance, leucine was used in the following experiments. C2C12 cells were pre-treated with leucine (0, 10 mM) for 2 h, followed by the treatment with T (0, 5 × 10^-7^ M) for 24 h. The cells were then collected for protein assay. As illustrated in Figure 6A, 6B and 6C, the co-treatment of T with leucine significantly increased the phosphorylation of mTOR and S6 compared with control cells (*P* < 0.01 and *P* < 0.01, respectively). In addition, the phosphorylation of mTOR and S6 was significantly higher in T+leucine group than in T group (*P* < 0.05 and *P* < 0.05, respectively) (Figure 6B and 6C), suggesting the further activation of mTORC1 by leucine. Similar to T treatment alone, T+leucine reduced the ratio of LC3-II/LC3-I (*P* < 0.01) (Figure 6D) and increased p62 expression (*P* < 0.01) (Figure 6E) compared with control cells, suggesting the inhibition of autophagy by T+leucine treatment. Moreover, the LC3-II/LC3-I ratio was significantly lower (*P* < 0.05) and p62 expression significantly higher (*P* < 0.01) in T+leucine group than in T group, indicating the enhanced effect of leucine on inhibiting autophagy. Similar to T treatment alone, T+leucine also induced a marked decrease in the integrated mtDNA/nDNA ratio (*P* < 0.01) (Figure 6F), suggesting the reduced integrated mtDNA copy number by T+leucine treatment. In addition, T+leucine caused a marked decrease in ATP production (*P* < 0.05) (Figure 6G) compared with control cells. These data suggested that T+leucine treatment reduced the number of integrated mitochondria and impaired mitochondrial function. But there was no obvious difference in the integrated mtDNA/nDNA ratio or ATP production between T and T+leucine groups. It is probably due to the already highly activated mTORC1 by T treatment.

We also evaluated plasma membrane GLUT4 expression and glucose uptake of C2C12 cells with treatments of T or T+leucine. As illustrated in Figure 6H and I, T+leucine or T treatment significantly decreased insulin-induced membrane GLUT4 levels and glucose uptake (*P* < 0.01 and *P* < 0.01, respectively). However, there was no marked difference between insulin+T and insulin+ T+leucine groups.

Insulin sensitivity was then investigated. Western blot analysis showed the significantly increased phosphorylation of Akt by insulin (*P* < 0.01), which was markedly reduced by T or T+leucine (*P* < 0.05 and *P* < 0.01, respectively) (Figure 6J). There was no apparent difference in p-Akt/Akt between insulin+T and insulin+ T+leucine groups.

In summary, these results suggested that both leucine and T activated mTORC1. As a result, T+leucine treatment impaired mitochondrial function, reduced insulin-stimulated GLUT4 translocation and glucose uptake, and induced insulin resistance. There was no apparent difference in these effects between T and T+leucine treatments.

### Effects of chloroquine (CQ) on T-treated C2C12 cells

**Figure 7:**
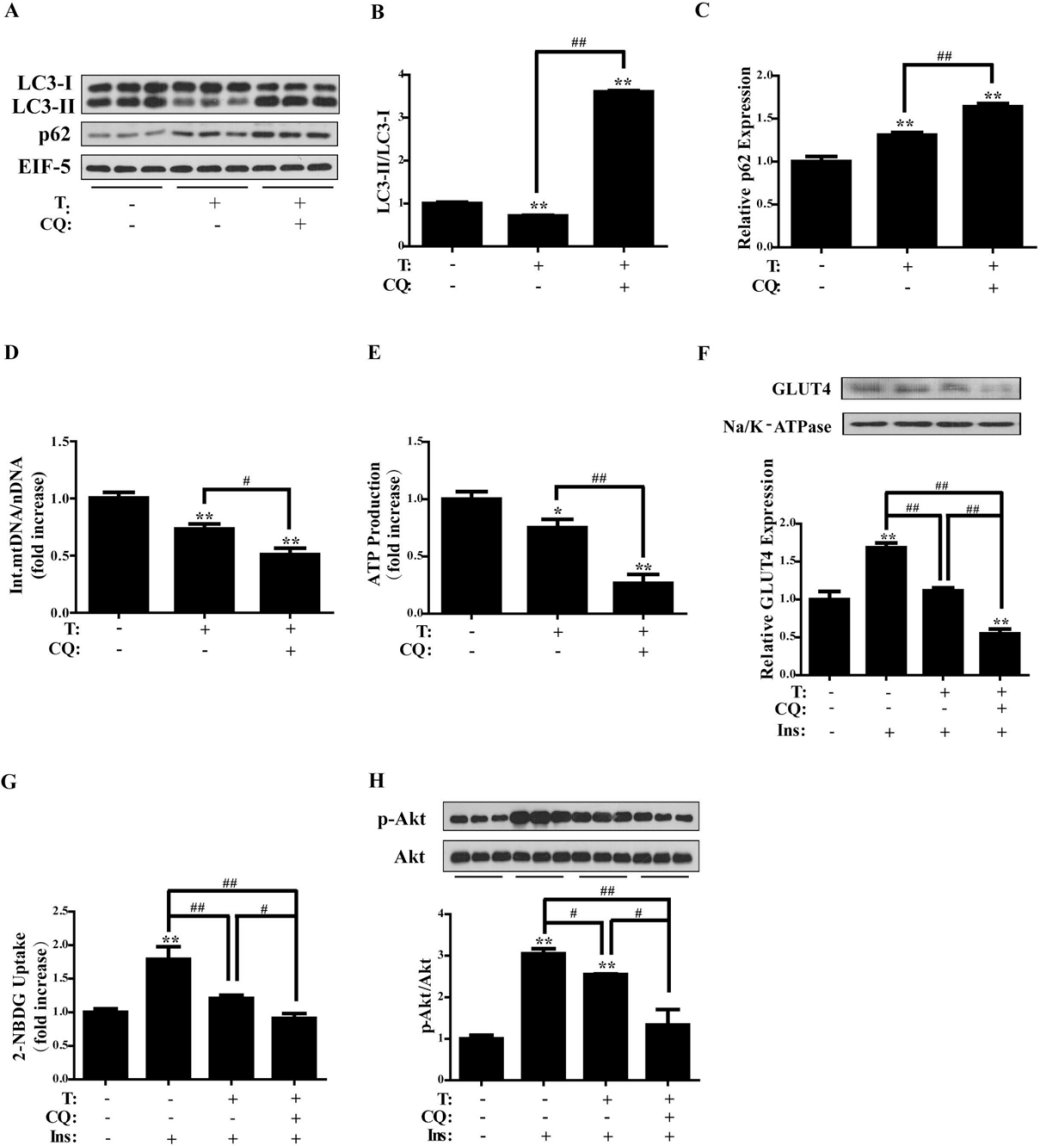
Effects of chloroquine on testosterone-treated C2C12 cells Differentiated C2C12 cells were pre-treated with chloroquine (0, 30 μM) for 2 h, followed by the treatment with T (0, 5 × 10^-7^ M) for 24 h. (**A**) Representative Western blots and densitometry quantification of (**B**) LC3-II/LC3-I ratio, and (**C**) p62. EIF-5 was used as a control. (**D**) Ratio of integrated mitochondrial to nuclear DNA number. (**E**) Normalized ATP production. (**F**) Representative Western blots and densitometry quantification of plasma membrane GLUT4. Na/K-ATPase served as a loading control of plasma membrane protein. (**G**) 2-NBDG glucose uptake assay. (**H**) Insulin-signaling assay. Data are expressed as mean ± SEM. ^*^P < 0.05, ^**^P < 0.01, vs control; ^#^*P* < 0.05, ^##^*P* < 0.01. *n* = 6/group. T: testosterone, CQ: chloroquine. GLUT4: glucose transporter 4.

CQ acts as a late-stage autophagy inhibitor by blocking autophagosome-lysosome fusion. To evaluate the role of autophagy in T-caused impairment, CQ was thus used. Cells were pre-treated with CQ (0, 30 μM) for 2 h, followed by the treatment with T (0, 5 × 10^-7^ M) for 24 h. Then the cells were collected for protein assay. Due to the mechanism by which CQ inhibits autophagy, treatment of T+CQ significantly increased the LC3-II/LC3-I ratio (*P* < 0.01) and p62 expression compared with control cells (*P* < 0.01) (Figure 7A, 7B, and 7C), indicating the inhibition of autophagy by CQ. As expected, the LC3-II/LC3-I ratio and p62 expression were significantly higher in T+CQ group than in T group (*P* < 0.01 and *P* < 0.01, respectively), suggesting the enhanced inhibition of autophagy by CQ.

The integrated mtDNA copy number was also measured. There was a marked decrease in the integrated mtDNA/nDNA ratio in T+CQ group compared with control and T groups (*P* < 0.01 and *P* < 0.05, respectively) (Figure 7D). Similar to T treatment alone, T+CQ treatment markedly decreased ATP production compared with control cells (*P* < 0.01) (Figure 7E). ATP production was also significantly lower in T+CQ group than in T group (*P* < 0.01). These results suggested that the enhanced inhibition of autophagy by CQ further damaged the mitochondria of the cells.

We also evaluated plasma membrane GLUT4 expression and glucose uptake of the cells with pre-treatment of CQ. As illustrated in Figure 7F and 7G, insulin+T and insulin+T+CQ treatments significantly decreased membrane GLUT4 levels and glucose uptake compared with insulin group (*P* < 0.01 and *P* < 0.01, respectively). Moreover, there was a significant decrease in membrane GLUT4 and glucose uptake in insulin+T+CQ compared with insulin+T group (*P* < 0.01 and *P* < 0.05, respectively) (Figure 7F and G), indicating that T+CQ further decreased insulin-stimulated GLUT translocation and glucose uptake.

Likewise, insulin sensitivity was investigated. Cells were pre-treated with CQ for 2 h and then treated with T for 24 h, followed by the treatment with insulin (0, 100 nM) for 5 min. Western blot analysis showed the significantly increased phosphorylation of Akt by insulin (*P* < 0.01), which was markedly reduced by T or T+CQ (*P* < 0.05 and *P* < 0.01, respectively) (Figure 7H). In addition, p-Akt/Akt ratio was significantly lower in insulin+T+CQ group than in insulin+T group (*P* < 0.05) (Figure 7H). These data suggested that CQ inhibited autophagy and therefore exacerbated T-induced insulin resistance in the cells.

Collectively, these results suggested that inhibition of autophagy by CQ further impaired mitochondria, reduced insulin-stimulated glucose uptake, and induced insulin resistance in the cells.

### Effects of the different treatments on mitochondrial metabolism

**Figure 8:**
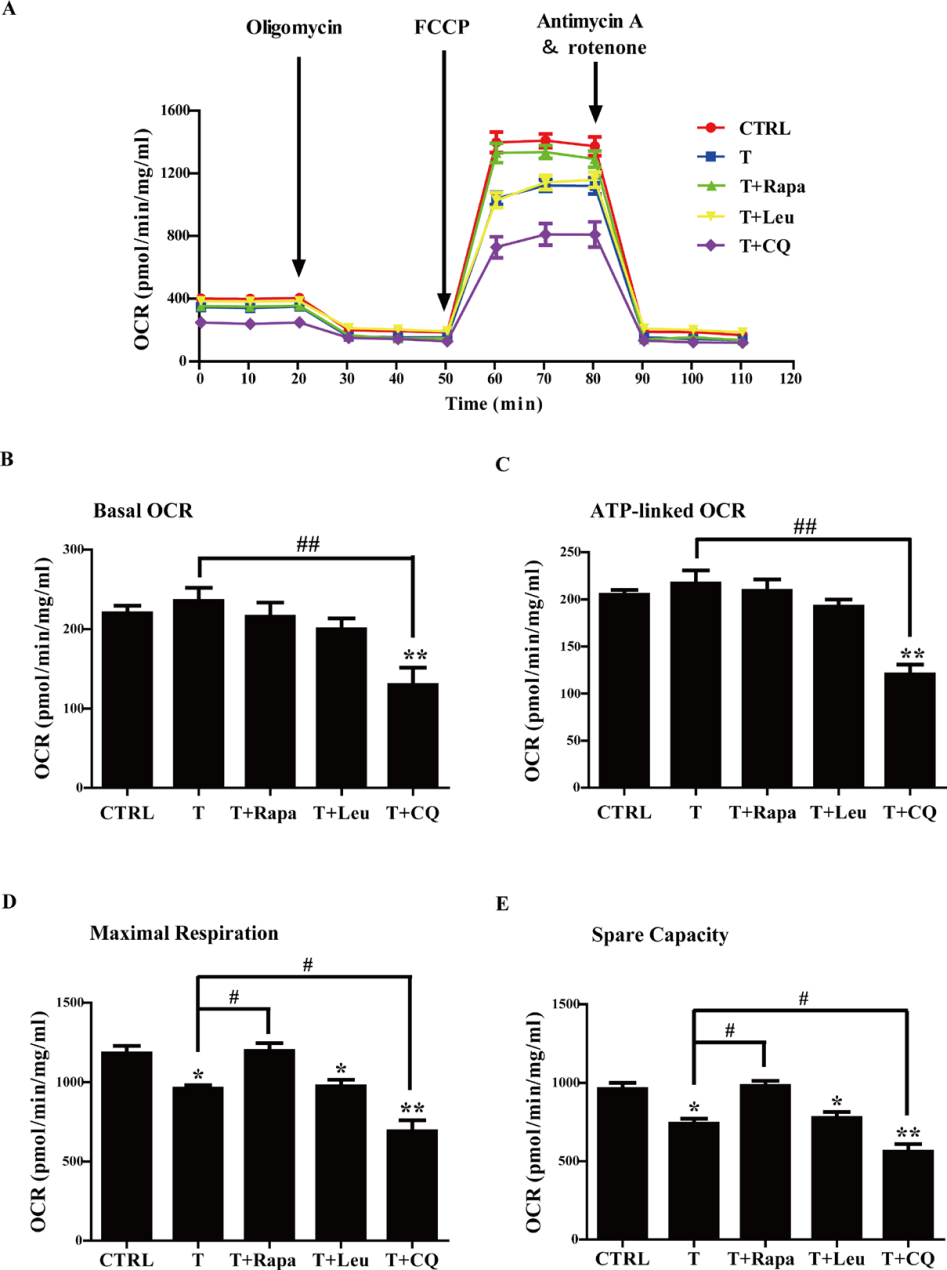
Oxygen consumption rate (OCR) in response to different treatments in C2C12 cells (**A**) Representative experiment to determine OCR in C2C12 cells in response to different treatments. Oxygen consumption was measured using a Seahorse XF24 analyzer, with additions of reagents at the indicated time points. (**B**) Quantification of the basal OCR. (**C**) Quantification of the ATP-linked OCR. (**D**) Quantification of maximal respiration OCR. (**E**) Quantification of spare capacity OCR. Data are expressed as mean ± SEM. ^*^P < 0.05, ^**^P < 0.01, vs control; ^#^*P* < 0.05, ^##^*P* < 0.01. *n* = 4/group. T: testosterone, Rapa: rapamycin, Leu: leucine, CQ: chloroquine.

Oxygen consumption rate (OCR) measurements are one of the preferred methods of evaluating mitochondrial function or dysfunction in cultured cells. We thus determined OCRs in response to different treatments. Differentiated C2C12 cells were treated with vehicle, T, T+rapamycin, T+leucine, or T+CQ, as described before. Cells treated with vehicle were defined as controls. Then the cells were sequentially treated with the ATP synthase inhibitor oligomycin, the mitochondrial oxidative phosphorylation uncoupler FCCP, and the combination of the electron-transport-chain inhibitors rotenone with antimycin A. The OCR, attributed to basal, ATP coupled, maximal respiration, and spare capacity, was therefore monitored. As illustrated in Figure 8A, B and C, there was no apparent difference in the basal or ATP-linked OCRs among control, T, T+rapamycin, and T+leucine groups. T+CQ treatment induced a significant decrease in both basal and ATP-linked OCRs compared with T and control groups (*P* < 0.01 and *P* < 0.01, respectively). The reduction in maximal respiration and spare capacity suggests the injured mitochondrial ETC. Moreover, the reduced spare capacity also indexes the decreased response to ATP demand. Treatments of T, T+leucine, and T+CQ markedly reduced the maximal respiration and spare capacity OCRs compared with controls (*P* < 0.05, *P* < 0.05, and *P* < 0.01, respectively) (Figure 8D and E). In addition, the maximal respiration and spare capacity OCRs were significantly lower in T+CQ group than in T group (*P* < 0.05 and *P* < 0.05, respectively), suggesting that reduced autophagy by CQ further impaired mitochondrial function. In contrast, the maximal respiration and spare capacity OCRs were significantly higher in T+rapamycin group than in T group (*P* < 0.05 and *P* < 0.05, respectively), implying that suppressing mTORC1 or inducing autophagy by rapamycin rescued the impaired mitochondrial function. There was no difference between T and T+leucine groups, indicating that up-regulating mTORC1 by leucine did not further worsen the impaired mitochondrial function. There were no changes in proton leak among the different treatments (data not shown), suggesting the intact mitochondrial electron transport chain (ETC) integrity in all the groups.

Collectively, these data indicated that T treatment injured mitochondrial ETC and reduced cellular responses to ATP demand. Inhibition of autophagy by CQ exacerbated T-impaired mitochondrial function of the cells. On the contrary, inhibition of mTORC1 or induction of autophagy by rapamycin restored T-induced mitochondrial impairment.

## DISCUSSION

A link between PCOS and IR was first highlighted by Burghen *et al.* in 1980 [12]. PCOS-related IR confers a substantially increased risk for T2D in these patients. Although it is generally agreed that there is a post-binding defect in insulin receptor signaling, the pathogenesis of IR in PCOS is incompletely understood. IR has been reported in skeletal muscle, adipose tissue, and ovaries in PCOS. Skeletal muscle is one of the classic insulin target tissues and IR in skeletal muscle stands as an early defect in the development of T2D. It has been reported that the pathogenesis of IR in skeletal muscle involves fat accumulation in the myocytes, decreased insulin-stimulated glucose uptake, reduced glycogen synthesis, mitochondrial defect, et al [13]. Androgen excess is a key feature of PCOS and androgens contribute to IR in PCOS. However, the role for hyperandrogenism in the development of skeletal muscle IR in PCOS remains largely unknown.

Hyperandrogenism occurs in about 60-80% of PCOS women. The association of hyperandrogenism and insulin resistance has been studied for decades. In males and females, the role for androgens in glucose metabolism appears to be different. Sato and colleagues have reported that DHEA administration improved insulin resistance and hyperglycemia in high-sucrose diet-induced obese male rats [14, 15] or streptozotocin (STZ)-induced male rats with type 1 diabetes mellitus [16]. In females, however, androgen promotes insulin resistance in the mice [17]. IR and compensatory hyperinsulinemia cause hyperandrogenemia in women [15] and androgen excess per se might also impair insulin action. It has been proposed that androgen excess in women may directly impair insulin action in adipose tissue and skeletal muscle [18]. Indeed, a systemic review has demonstrated a significantly higher risk of developing T2D in women with elevated T concentration [19]. Androgen administration to healthy premenopausal women caused insulin resistance. Moreover, Moghetti and colleagues have shown that antiandrogen treatment partially reversed the peripheral insulin resistance associated with hyperandrogenism in women [20]. *In vitro*, chronic T treatment induced IR in subcutaneous adipocytes of premenopausal women [21]. In animal models, prenatal and neonatal exposure to androgens resulted in IR [18]. In ovariectomized rats, administration of T caused skeletal muscle IR [22]. The underlying mechanisms, however, remain unknown. Previously, we and others have shown that injection of DHEA into the female mice at 25-day old for 20 consecutive days caused IR in the C57BL/6 strain based on insulin tolerance test (ITT) [10] and in the BALB/c strain based on HOMA index [23], respectively. In the present work, we further confirmed that DHEA-induced PCOS mice exhibited whole-body insulin resistance by fasting serum insulin measurement and HOMA-IR evaluation. In addition, we also demonstrated skeletal muscle IR in DHEA mice by insulin-signaling assay. Our findings suggest that exposure to excess androgen impairs insulin action in skeletal muscle in the prepubertal female mice.

Insulin activation of Akt is essential for the regulation of cellular glucose uptake. The p-Akt/Akt ratio is often used to evaluate insulin sensitivity of tissues. The significant attenuation of insulin-stimulated phosphorylation of Akt has been reported in skeletal muscle of T2D. In addition, Akt/mTOR signaling pathway is involved in insulin resistance. In the liver and skeletal muscle of obese and high-fat-fed rodents, mTORC1 is highly active [24, 25]. Guridi et al. have recently shown that alterations of mTORC1 signaling in the skeletal muscle differentially affect whole-body metabolism [26]. In addition, autophagy plays a critical role for myofiber maintenance and its activation is crucial to avoid accumulation of toxic proteins and dysfunctional organelles [27]. Yaba et al. have reported that DHEA increased mTOR expression in mouse ovaries in DHEA-induced PCOS mice [28]. But it remains unknown whether mTOR signaling as well as mTORC1-regulated autophagy changes in skeletal muscle of DHEA-treated mice. In the present study, we showed the decreased phosphorylation of Akt after insulin administration and activated mTORC1 signaling in skeletal muscle of the DHEA mice. Activation of mTORC1 suppresses autophagy. Indeed, reduced autophagy was observed in skeletal muscle of DHEA mice in the present work, as evidenced from the reduced LC3-II/LC3-I ratio and the increased p62 expression. Autophagy is an important regulator of insulin signaling. Loss of autophagy is a critical component of defective insulin action seen in obesity [29]. But the change of autophagy with IR seems to be tissue specific. Moller and colleagues have reported the decreased autophagy-related gene expression in skeletal muscle from T2D patients [30]. However, the attenuated mTOR signaling and enhanced autophagy were shown in adipocytes from obese patients with T2D [31]. Based on the results from the present study, we proposed that changes of mTORC1 and mTORC1-regulated autophagy may contribute to skeletal muscle IR. We next investigated the underlying mechanism.

Allemand et al. have reported a synergistic interaction between T and insulin [32] by using primary rat myotubes. To explore the mechanism by which DHEA induces skeletal muscle insulin resistance in prepubertal female mice, C2C12 cells were cultured and treated with androgen. Circulating DHEA is converted to T by 3b-hydroxysteroid dehydrogenase (HSD) and 17b-HSD *in vivo*, and T in turn is converted to estrogens by aromatase cytochrome *P*-450 (P450arom). It has been reported that these enzymes are expressed at the mRNA and protein levels in skeletal muscle or skeletal muscle cells [33]. Skeletal muscles are capable of locally synthesizing T and estradiol from circulating DHEA. In the present study, we found that T levels were significantly higher in the skeletal muscle DHEA mice than in controls, whereas estradiol levels were similar between these two groups of animals (Supplementary Figure 1). T was thus used in the cell experiments. Results from the experiments with T at a series of concentrations revealed that T at 5 × 10^-7^ M increased mTOR activation and decreased autophagy, which mimicked the *in vivo* situation. In addition, compared with control cells, T did not seem to have any apparent effect on the phosphorylation of Akt in the absence of insulin. In DHEA mice, the level of p-Akt was similar to that in control animals without insulin administration. These data suggest that excess androgen may up-regulate mTORC1 activation in an Akt-independent pathway. In addition, treatment of C2C12 cells with T at 5 × 10^-7^ M impaired mitochondrial function and reduced insulin-stimulated GLUT4 translocation and glucose uptake, leading to insulin resistance.

It is generally agreed that insulin resistance in skeletal muscle is linked to mitochondrial dysfunction. Several lines of evidence support a reduction in mitochondrial number in insulin resistant skeletal muscle. In the present study, we have also found the decreased integrated mtDNA copy number and increased abnormal mitochondria in skeletal muscle of DHEA mice, as evidenced by the reduced ratio of integrated mtDNA to nDNA and results from TEM of the skeletal muscle tissues. Impaired mitochondria are likely to lead to the damaged mitochondrial function. Indeed, ATP production was reduced in skeletal muscle of DHEA mice, indicating the impaired mitochondrial function in skeletal muscle of DHEA mice. Meanwhile, the results from OCR measurements further suggested that T treatment impaired mitochondrial function in C2C12 cells. Together, we showed the increased abnormal mitochondria and the impaired mitochondrial function in skeletal muscle of DHEA mice. Moreover, our data demonstrated that treatment of C2C12 cells with high dose T reduced autophagy and damaged mitochondrial function. Reduced autophagy and autophagy-induced mitochondrial impairment are thus likely to contribute to skeletal muscle IR in DHEA mice.

In addition to mitochondrial dysfunction, impaired glucose uptake also plays a critical role in the development of insulin resistance in skeletal muscle. Decreased insulin-stimulated glucose transport in skeletal muscle has been shown to be a major contributing factor to IR in patients with T2D and obesity. Additionally, it has been reported that T reduced insulin-stimulated glucose uptake in cultured female adipocytes [21] and endometrial cells [34]. In the present work, we demonstrated that T decreased insulin-stimulated GLUT4 translocation and glucose uptake in C2C12 myotubes, which promoted insulin resistance in these cells.

To further address the role for mTORC1 and its regulated autophagy in T-induced skeletal muscle IR, rapamycin, leucine, or CQ was used in combination with T. Our data showed that inhibition of mTORC1 or induction of autophagy by rapamycin improved mitochondrial function and increased glucose uptake, finally leading to the up-regulation of insulin sensitivity in T-treated C2C12 cells. On the contrary, inhibition of autophagy by CQ further exacerbated T-impaired mitochondrial function and insulin-stimulated glucose uptake, causing insulin resistance. Meanwhile, results from OCR measurements have also indicated that inhibition of autophagy by CQ impaired mitochondrial function. In contrast, T-impaired mitochondrial function can be restored by rapamycin co-treatment. Leucine activates mTOR. But the treatment of T+leucine did not seem to further worsen T-caused impairment, possibly due to the high level of mTORC1 phosphorylation already induced by T. Consistent with our results, Li *et al.* has recently shown that Sesn2-induced autophagy contributed to restore the impaired insulin signaling in C2C12 myotubes [35]. Collectively, our data show that reduced autophagy caused mitochondrial impairment and decreased glucose uptake, leading to insulin resistance in C2C12 cells. These results suggest a crucial role of autophagy in the pathogenesis of skeletal muscle IR.

**Figure 9:**
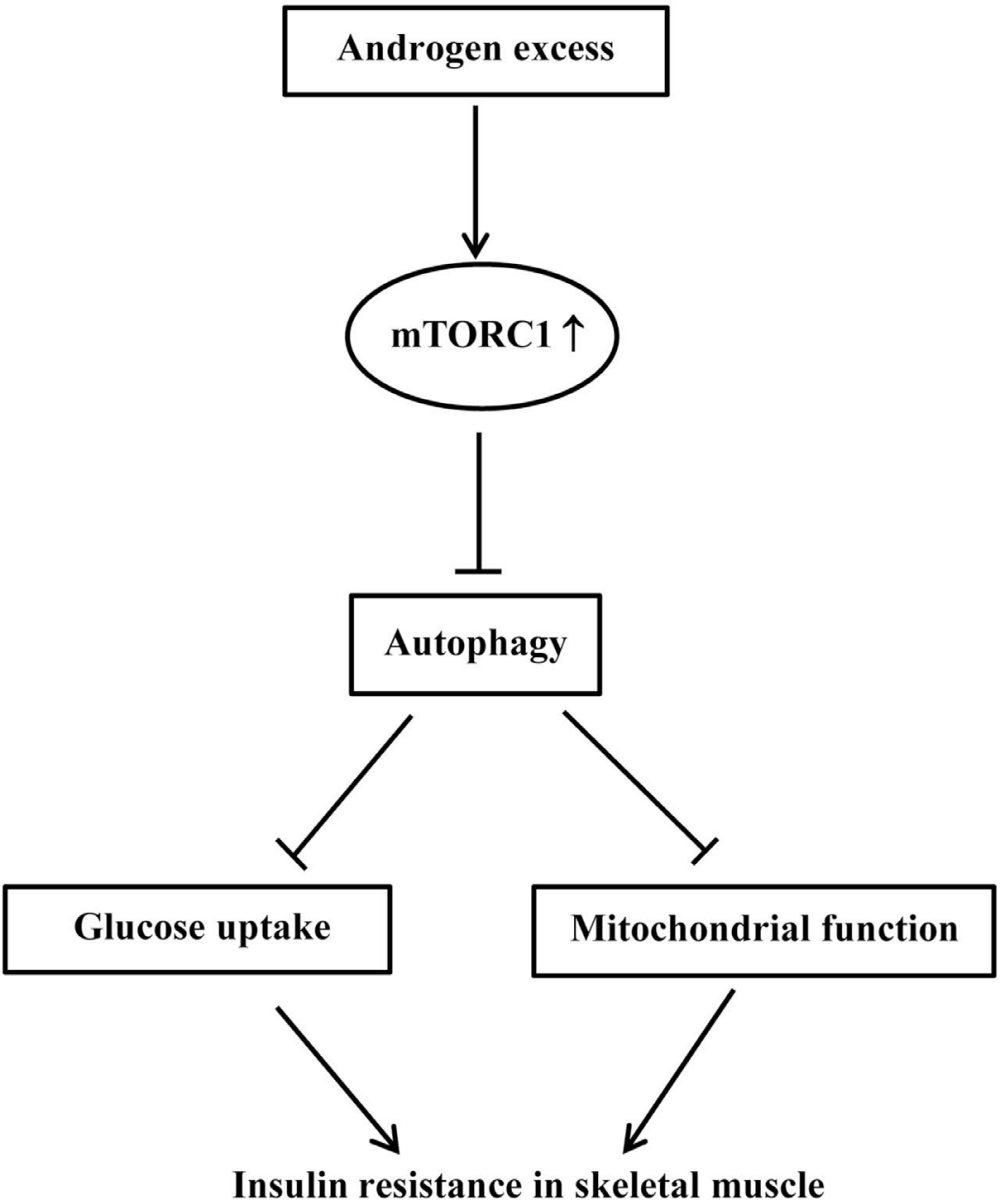
Schematic overview of mTORC1-autophagy pathway in excess androgen-induced skeletal muscle insulin resistance

In conclusion, we present here that early exposure to androgen excess in prepubertal female mice induces whole-body and skeletal muscle IR. Dysregulation of mTORC1-autophagy pathway led to mitochondrial impairment and decreased glucose uptake, contributing to hyperandrogenism-induced skeletal muscle IR. The proposed mechanism by which hyperandrogenism induces skeletal muscle IR is shown in Figure 9. Our findings indicate the importance of mTORC1-autophagy pathway in insulin resistance of skeletal muscle in PCOS. These data would help to better understand the role of hyperandrogenism and the underlying mechanism in the pathogenesis of skeletal muscle IR in PCOS.

## MATERIALS AND METHODS

### Animals and treatments

Female C57BL/6 mice (age 21 days) were purchased from the Animal facility of Peking University Health Science Center. All animals were bred, housed locally at 22°C ± 2°C, and were acclimated to standard laboratory conditions (12L:12D cycle) with free access to rodent food and water. At postnatal day 25, the mice of comparable weights were randomly divided into two groups (*n* = 12/group): group 1, control group. The mice were injected (s.c.) daily with sesame oil (0.1 ml/100g body weight); group 2, DHEA group. Mice were injected (s.c.) daily with DHEA (6 mg/100g body weight) dissolved in 0.1 ml of sesame oil. The animals were treated for 20 days and then six mice of each group were subjected to insulin-signaling assay. The other mice were killed and tissue samples were collected. All experimental protocols were approved by the Ethics Committee for Animal Experimentation of the Faculty of Medicine of Peking University Health Science Center in accordance with the Guide for the Care and Use of Laboratory Animals published by the US National Institutes of Health. DHEA and sesame oil were purchased from Sigma-Aldrich (St-Louis, MO, USA). Additional information about the animal model used in the study can be found in Supplementary Figure 2 and in our previous work [10].

### Insulin-signaling assay

After the treatments for 20 days, three mice from each group were fasted overnight and then injected intraperitoneally with 1.0 IU/kg body weight of insulin (insulin stimulation) or 0.9% saline (basal level) to measure the insulin signaling response. Skeletal muscles from the hind limbs (quadriceps muscle) were collected at 15 min after insulin injection and snap frozen in liquid nitrogen. The protein expression of total Akt and phosphorylated Akt (Ser473) was determined by Western blot.

### Tissue collection

After the treatments for 20 days, six mice from each group were fasted overnight and were then deeply anesthetized via an intraperitoneal injection of a mixture of ketamine hydrochloride and xylazine. Blood samples were collected from inferior vena cava. Skeletal muscles from the hind limbs (quadriceps muscle) were dissected, snap frozen in liquid nitrogen, and then kept at -80°C for protein assay.

### HOMA-IR

For estimation of whole-body insulin resistance, the homeostasis model assessment index of insulin resistance (HOMA-IR) was calculated according to the following equation: HOMA-IR  =fasting insulin (mIU/L) × fasting glucose (mmol/L)/22.5. Serum insulin levels were determined with ^125^I-labeled insulin radioimmunoassay kits (Beijing North Institute of Biological Technology). The within-assay and between-assay variabilities were 10 and 15%, respectively.

### Measurement of Serum Sex Steroids

Skeletal muscle testosterone and estradiol levels were determined with ^125^I-labeled radioimmunoassay kits (Beijing North Institute of Biological Technology). The withinassay and between-assay variabilities were 10% and 15%, respectively.

### Transmission electron microscopy (TEM)

The tissues of skeletal muscle were fixed in 3% glutaraldehyde and 1% osmium, dehydrated through graded concentrations of acetone solutions, and embedded in Epon812. Thin sections (70 nm) were cut using an ultramicrotome (Leica UC7, Germany), double stained with uranyl acetate and lead citrate, and analyzed using a JEM-1230 electron microscope (JEOL-Ltd, Tokyo, Japan). Quantitative analysis of the percentage of abnormal mitochondria was performed. Ten randomly selected section of each specimen was photographed at ×55,000 magnification. Ten to twenty micrographs per sample were randomly selected. The number of abnormal mitochondria and total mitochondria was counted to calculate the percentage of abnormal mitochondria.

### Determination of ATP content

ATP contents of skeletal muscle tissues or cultured cells were measured using a commercially available kit (ATP-Lite Assay Kit, Vigorous Biotechnology, Beijing, China). The ATP content was measured using a Luminometer (Turner BioSystems, Sunnyvale, USA), normalized by the protein concentration (nmol/mg protein) in the same sample, and presented as the fold of the control.

### Integrated mitochondrial DNA (mtDNA) copy number

Total DNA was isolated from skeletal muscle tissues using phenol/chloroform/isoamyl alcohol (25:24:1) followed by ethanol precipitation. Integrated mtDNA (ND4, forward primer 5′-ATCGCACATGGCCTCACATC-3′; reverse primer 5′-TGTGTGTGAGGGTTGGAGGT-3′) and nuclear DNA (nDNA) (Lpl, forward primer 5′-GGATGGACGGTAAGAGTGATTC-3′; reverse primer 5′-ATCCAAGGGTAGCAGACAGGT-3′) were amplified and quantified using real-time PCR. Real-time PCR was performed using fluorescent SYBR Green according to the manufacturer’s instructions (Invitrogen, Carlsbad, CA, USA). The integrated mtDNA copy number was calculated by normalizing ND4 copies to Lpl copies in the same sample.

### Cell culture and treatments

C2C12 mouse myoblasts from China Infrastructure of Cell Line Resource (Beijing, China) were cultured in Dulbecco’s Modified Eagle’s Medium (DMEM) containing 4500 mg/L glucose and supplemented with 10% fetal bovine serum (FBS), 4 mM glutamine, and 100U/mL penicillin/ streptomycin, in a humidified 5% CO_2_ atmosphere at 37°C. Cells were seeded overnight and grown to confluence with growth media changed every two days. At 80% confluence, the cells were allowed to differentiate by replacing growth media with DMEM supplemented with 2% horse serum and 100U/mL penicillin/streptomycin for 7 days. The fully differentiated C2C12 cells were used for the different treatments.

### Treatments of C2C12 cells with testosterone (T)

The differentiated C2C12 cells were treated with different concentrations of T (Sigma-Aldrich, St-Louis, MO, USA) (0, 5 × 10^-9^, 5 × 10^-8^, 5 × 10^-7^, and 5 × 10^-6^M) in triplicate at 37°C in an atmosphere of 5% CO_2_:95% air. After 24 h, cells were collected for protein assay.

### Extraction of plasma membrane protein

The plasma membrane protein of skeletal muscle tissue or cells was isolated with Minute™ plasma membrane protein isolation kit (Invent Biotechnologies, Inc., Plymouth, MN, USA) according to the manufacturer’s instructions.

### 2-NBDG glucose uptake assays

Glucose uptake was measured using a commercially available kit from Invitrogen (Carlsbad, CA, USA). Briefly, fully differentiated C2C12 cells were treated with or without testosterone (5×10^-7^ M) for 24 h, followed by the treatment with 60 μM of 2-(N-(7-nitrobenz-2-oxa-1,3-diazol-4-yl)amino)-2-deoxyglucose (2-NBDG) in the presence or absence of 100 nM of insulin in PBS and incubated at 37°C for 1 h. The supernatant was then removed and the cells were rinsed twice with PBS. Fluorescent intensity was recorded using a Varioskan^R^ Flash microplate reader (Thermo Scientific, Finland) at excitation and emission wavelengths of 485 nm and 535 nm, respectively.

### Western blot analysis

Western blot was performed as described previously [10]. Briefly, proteins from tissues or cells were extracted and quantified. Aliquots of 10 μg total protein of each sample were separated by 8%-15% SDS-PAGE and transferred to nitrocellulose membrane for all the proteins except for LC3, where PVDF membranes were used for LC3 signal. The membrane was blocked in 5% skimmed milk-TBST solution for 1 hour at room temperature. The membrane was probed with primary antibodies at 4°C overnight. Antibodies to Akt, phosphor-Akt at Ser473 (p-Akt), mTOR, phosphor-mTOR at Ser2448 (p-mTOR), LC3A/B, p62, ribosomal protein S6, ribosomal protein phosphor-S6 at Ser235/236 (p-S6), and Na/K-ATPase were purchased from Cell Signaling Technology. The antibodies to glucose transporter 4 (GLUT4) (H-61) and EIF-5 were bought from Santa Cruz Biotechnology (CA, USA). EIF-5 was measured as an internal control of total protein. Na/K-ATPase was used as a loading control of plasma membrane protein. The membrane was washed for 5 min with TBS-T buffer for 5 times and then incubated with a horseradish peroxide-conjugated secondary antibody at room temperature for 1 h. After washing, the membrane was developed with ECL Reagent (Millipore, USA) and exposed to Kodak XBT-1 film. Protein expression level was quantified with Image J (NIH) software.

### Insulin-signaling assay in the cultured cells

The differentiated C2C12 cells were treated with testosterone (0, 5 × 10^-7^ M) or the combination of testosterone and other reagents for 24 h, followed by the treatment with insulin (0, 100 nM) for 5 min. Then the cells were collected for protein assay. The protein expression of total Akt and phosphorylated Akt (Ser473) was determined by Western blot.

### Oxygen consumption assay

Oxygen consumption was measured using a commercially available kit from Seahorse Bioscience (Seahorse Bioscience Inc., North Billerica, MA, USA). Oxygen consumption rates (OCRs) were measured using the XF-24 Extracellular Flux Analyzer (Seahorse Bioscience). The protocol was performed as reported with some modifications [36]. In brief, 5 × 10^5^ cells were seeded per well in 24-well XF microplates. When cells were differentiated into mature myotubes, they were treated with 0, T, and the combination of T with other reagents. After different treatments, cell culture medium was replaced with XF Assay Medium supplemented with 25 mM glucose, 1 mM pyruvate and 4 mM glutamine, and incubated in a CO_2_-free incubator at 37°C for 1 h for temperature and pH equilibration. The baseline oxygen consumption rate (OCR) was measured. The wells were then injected sequentially with oligomycin (1 µM) to measure the ATP-link OCR, oxidative phosphorylation uncoupler carbonyl cyanide-4-(trifluoromethoxy) phenylhydrazone (FCCP, 1 µM) to determine maximal respiration, and rotenone (0.5 µM) and antimycin A (0.5 µM) to determine non-mitochondrial respiration. The treatments were performed in quadruplicate and each experiment was repeated 3 times. OCRs were normalized by the protein concentrations.

### Statistical analysis

The values were presented as mean ± SEM. Statistical analysis was performed using GraphPad Prism 5.0 software. Effects of the treatments were analyzed by unpaired *t*-test for comparisons between two groups and by one-way analysis of variance (ANOVA) followed by Bonferroni’s posttest for multiple comparisons. *P* < 0.05 was considered statistically significant.

## SUPPLEMENTARY MATERIALS FIGURES



## Abbreviations

2-NBDG: 2-(N-(7-nitrobenz-2-oxa-1,3-diazol-4-yl)amino)-2-deoxyglucose
Akt: protein kinase B
CQ: chloroquine
DHEA: dehydroepiandrosterone
ETC: electron transport chain
FCCP: carbonyl cyanide-4-(trifluoromethoxy) phenylhydrazone
HOMA-IR: homeostasis model assessment index of insulin resistance
IR: insulin resistance
mTORC1: mammalian target of rapamycin complex 1
OCR: oxygen consumption rate
PCOS: polycystic ovary syndrome
T: testosterone
