# Supplementary material for: Dehydroepiandrosterone-induced activation of mTORC1 and inhibition of autophagy contribute to skeletal muscle insulin resistance in a mouse model of polycystic ovary syndrome
Source: Oncotarget. 2018 Jan 12;9(15):11905–21. doi: 10.18632/oncotarget.24190 (PMC5844717; doi:10.18632/oncotarget.24190)
Supplement: Supplementary file 1 [file oncotarget-09-11905-s001.pdf]

Dehydroepiandrosterone-induced activation of mTORC1 and inhibition of autophagy contribute to skeletal muscle insulin resistance in a mouse model of polycystic ovary syndrome

SUPPLEMENTARY MATERIALS

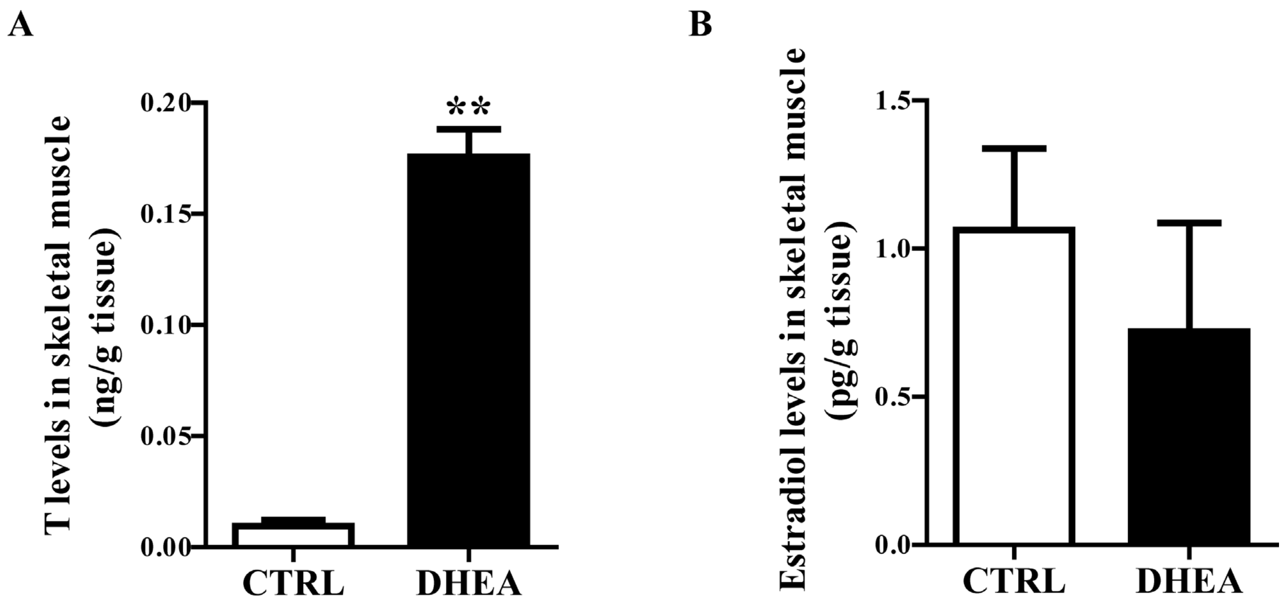

Supplementary Figure 1: Levels of sex steroid hormones in the skeletal muscle of the mice. (**A**) Testosterone levels in skeletal muscle. (**B**) Estradiol levels in skeletal muscle. *n* = 6/group. T: testosterone. Data are expressed as mean ± SEM. $^{**}P < 0.01$, vs control.

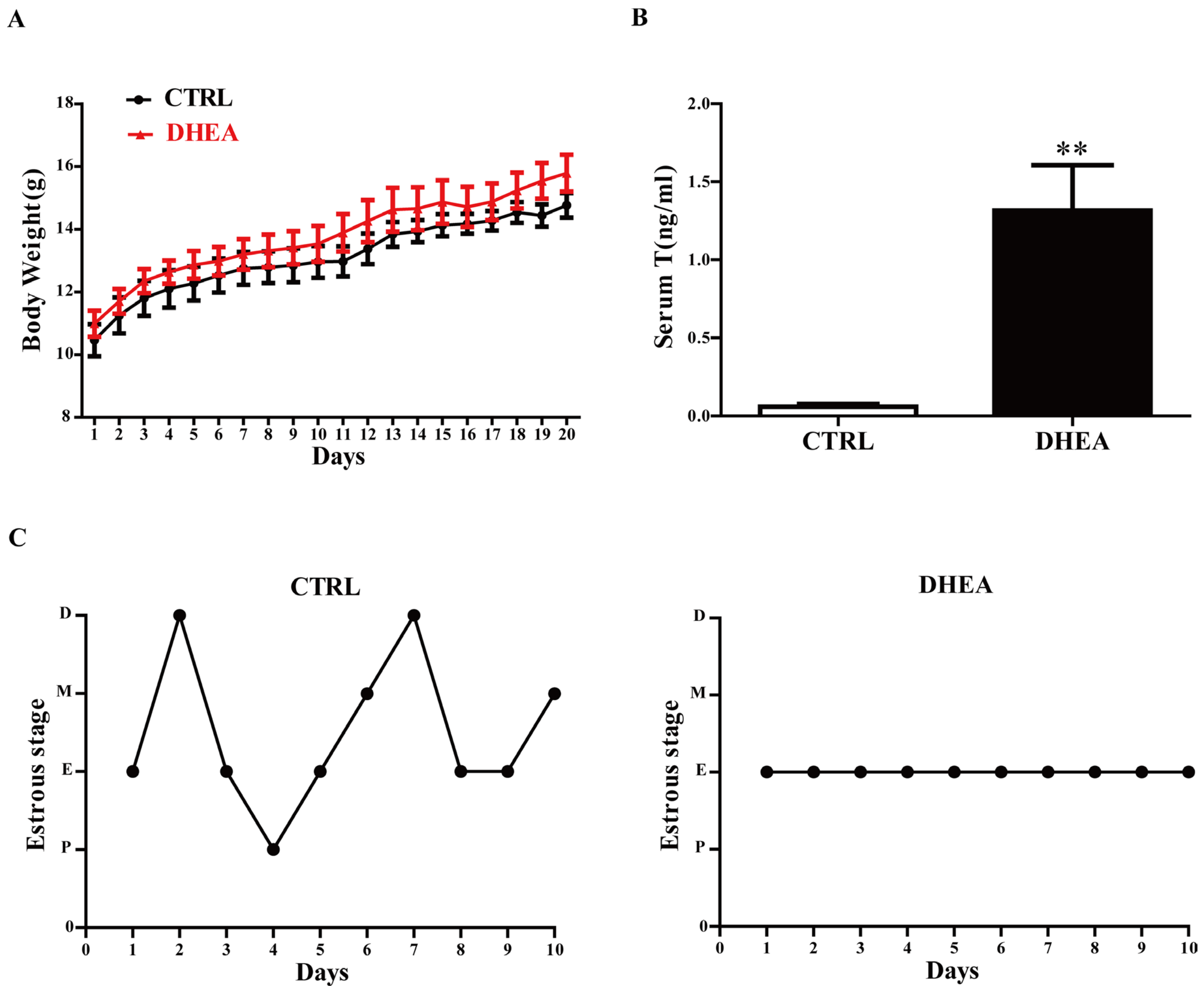

Supplementary Figure 2: Body weight, serum testosterone levels, and estrous cycle in the mice. (**A**) Body weight (**B**) Serum testosterone levels (**C**) Representative estrous cycle of one mouse from each group. $n = 6$/group. T: testosterone. Data are expressed as mean ± SEM. $^{**}P < 0.01$, vs control.
